# Demographic, multi-morbidity and genetic impact on myocardial involvement and its recovery from COVID-19: protocol design of COVID-HEART—a UK, multicentre, observational study

**DOI:** 10.1186/s12968-021-00752-1

**Published:** 2021-06-10

**Authors:** Miroslawa Gorecka, Gerry P. McCann, Colin Berry, Vanessa M. Ferreira, James C. Moon, Christopher A. Miller, Amedeo Chiribiri, Sanjay Prasad, Marc R. Dweck, Chiara Bucciarelli-Ducci, Dana Dawson, Marianna Fontana, Peter W. Macfarlane, Alex McConnachie, Stefan Neubauer, John P. Greenwood, the COVID-HEART investigators, Peter Swoboda, Richard Steeds, Timothy Fairbairn, Andrew Flett, Thomas Green, Graham Cole, Adam McDiarmid, Nicholas Bunce, Prathap Kanagala, Nicholas Bellenger, Tishi Ninan, Khaled Alfakih, James Moon

**Affiliations:** 1 Institute of Cardiovascular and Metabolic Medicine, University of Leeds, Leeds, LS2 9JT UK; 2 University of Leicester and The NIHR Leicester Biomedical Research Centre, Glenfield Hospital, Leicester, UK; 3 Institute of Cardiovascular and Medical Sciences and British Heart Foundation Glasgow Cardiovascular Research Centre, University of Glasgow, Glasgow, UK; 4 Division of Cardiovascular Medicine, Radcliffe Department of Medicine, Oxford Centre for Clinical Magnetic Resonance Research, British Heart Foundation Centre of Research Excellence, Oxford NIHR Biomedical Research Centre, University of Oxford, Oxford, UK; 5 Institute of Cardiovascular Science, University College London, London, UK; 6 Division of Cardiovascular Sciences, School of Medical Sciences, Faculty of Biology, Medicine and Health, University of Manchester, Manchester, UK; 7 School of Biomedical Engineering and Imaging Sciences, King’s College London, BHF Centre of Excellence and the NIHR Biomedical Research Centre at Guy’s and St. Thomas’ NHS Foundation Trust, The Rayne Institute, St. Thomas’ Hospital, London, UK; 8 National Heart and Lung Institute, Imperial College, London, UK; 9 University of Edinburgh and British Heart Foundation Centre for Cardiovascular Science, Edinburgh, UK; 10 Bristol Heart Institute, Bristol NIHR Cardiovascular Research Centre, University of Bristol and University Hospitals Bristol and Weston NHS Trust, Bristol, UK; 11 Department of Cardiology, Aberdeen Cardiovascular and Diabetes Centre, Aberdeen Royal Infirmary and University of Aberdeen, Aberdeen, UK; 12 Division of Medicine, Royal Free Hospital, University College London, London, UK; 13 Electrocardiology Core Laboratory, Institute of Health and Wellbeing, University of Glasgow, Glasgow, UK; 14 Robertson Centre for Biostatistics, Institute of Health and Wellbeing, University of Glasgow, Glasgow, UK; 15 Pinderfields Hospital, Wakefield, UK; 16 Queen Elizabeth Hospital, Birmingham, UK; 17 Liverpool Heart and Chest Hospital, Liverpool, UK; 18 Southampton General Hospital, Southampton, UK; 19 Northumbria NHS Trust, Ashington, UK; 20 Hammersmith Hospital, London, UK; 21 Freeman Hospital, Newcastle Upon Tyne, UK; 22 St Georges Hospital, London, UK; 23 Aintree University Hospital, Liverpool, UK; 24 The Royal Devon and Exeter Hospital, Exeter, UK; 25 Swansea Bay University Hospital, Port Talbot, UK; 26 Lewisham University Hospital, London, UK

**Keywords:** COVID-19, Coronavirus, Cardiovascular magnetic resonance, Myocarditis, Myopericarditis, Myocardial infarction, myocardial injury, Myocardial inflammation, Myocardial repair, Cardiovascular disease

## Abstract

**Background:**

Although coronavirus disease 2019 (COVID-19) is primarily a respiratory illness, myocardial injury is increasingly reported and associated with adverse outcomes. However, the pathophysiology, extent of myocardial injury and clinical significance remains unclear.

**Methods:**

COVID-HEART is a UK, multicentre, prospective, observational, longitudinal cohort study of patients with confirmed COVID-19 and elevated troponin (sex-specific > 99th centile). Baseline assessment will be whilst recovering in-hospital or recently discharged, and include cardiovascular magnetic resonance (CMR) imaging, quality of life (QoL) assessments, electrocardiogram (ECG), serum biomarkers and genetics. Assessment at 6-months includes repeat CMR, QoL assessments and 6-min walk test (6MWT). The CMR protocol includes cine imaging, T1/T2 mapping, aortic distensibility, late gadolinium enhancement (LGE), and adenosine stress myocardial perfusion imaging in selected patients. The main objectives of the study are to: (1) characterise the extent and nature of myocardial involvement in COVID-19 patients with an elevated troponin, (2) assess how cardiac involvement and clinical outcome associate with recognised risk factors for mortality (age, sex, ethnicity and comorbidities) and genetic factors, (3) evaluate if differences in myocardial recovery at 6 months are dependent on demographics, genetics and comorbidities, (4) understand the impact of recovery status at 6 months on patient-reported QoL and functional capacity.

**Discussion:**

COVID-HEART will provide detailed characterisation of cardiac involvement, and its repair and recovery in relation to comorbidity, genetics, patient-reported QoL measures and functional capacity.

*Clinical Trial registration:* ISRCTN 58667920. Registered 04 August 2020.

## Background

The coronavirus disease 2019 (COVID-19) severe acute respiratory syndrome coronavirus 2 (SARS-CoV-2) pandemic [1] was initially recognised as a primarily respiratory illness with a severe acute respiratory syndrome, but it is now known that it can affect multiple organs with a wide spectrum of disease severity [2, 3].

The direct cardiovascular impact of COVID-19 is three-fold. Firstly, underlying cardiovascular disease (CVD) can predispose patients to the infection and is associated with an increased illness severity. Secondly, COVID-19 may exacerbate underlying cardiovascular co-morbidities leading to symptom destabilisation and potentially acute admission to hospital. Thirdly, de novo cardiac complications of COVID-19 may occur [4], with SARS-CoV-2 infection being implicated in acute myocarditis, pericarditis, prothrombotic complications, left ventricular (LV) and/or right ventricular (RV) dysfunction, arrhythmia and ischaemic sequelae in the presence or absence of underlying coronary artery disease (CAD) [1, 4–6].

Multiple studies have demonstrated that myocardial injury, characterised by elevation in serum cardiac biomarkers, is common in COVID-19. The prevalence of myocardial injury, however, is variably reported, with early studies quoting a prevalence of 8–12% in hospitalised patients, whilst more recent data suggest that it is possibly much higher [4, 7]. Subsequently, a larger multicentre study of 305 patients identified an elevated troponin in more than half of the hospitalised patients [8]. A recent German cohort study reported imaging evidence of myocardial injury defined by cardiovascular magnetic resonance (CMR) in over 70%, although whether this was pre-existing or consequence was unclear with single time-point imaging [9]. The underlying mechanism of cardiac injury and troponin elevation remains unclear, but there are several potential explanations. Troponin T release is commonly seen in critically ill patients because of oxygen supply–demand mismatch and cytokine release, acute heart failure, RV strain (secondary to pneumonia, hypoxia and increased pulmonary artery pressure) and acute arrhythmia [10–12]. Furthermore, the acute-phase response to severe coronavirus infection can lead to coagulopathy and microvascular thrombosis, for example in the pulmonary and coronary circulations [13, 14]. Pre-existing coronary risk factors, such as hypertension and diabetes also predispose patients with COVID-19 to myocardial infarction in the acute setting, possibly implicating it as another potential disease mechanism [15]. On a cellular level, however, direct myocardial injury may occur due to viral angiotensin-converting enzyme-2 transmembrane receptor mediated damage, microvascular dysfunction or increased vessel wall permeability [16].

Troponin elevation in hospitalised COVID-19 patients is associated with higher morbidity and increased short-term mortality [4, 8, 17–21], particularly in those who also have underlying CVD [16]. Those with CV comorbidities (diabetes, hypertension, heart failure or CAD/peripheral artery disease) are heavily over-represented in both severe COVID-19 and mortality [22, 23].

**Fig. 1:**
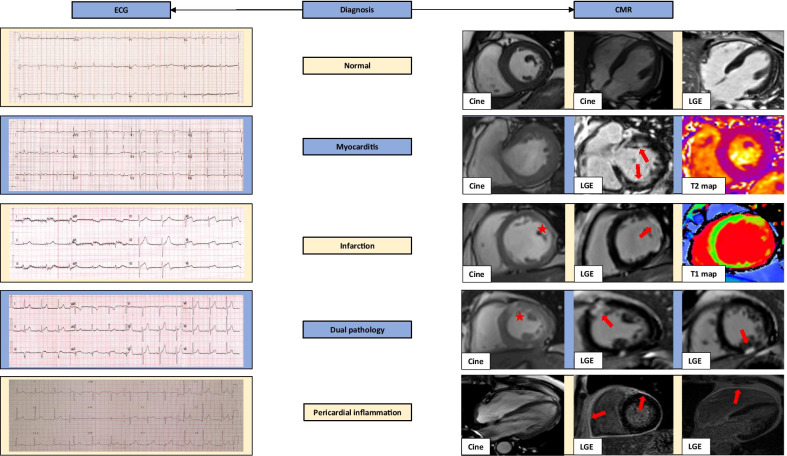
Electrocardiogram (ECG) and cardiovascular magnetic resonance (CMR) examples in troponin positive COVID-19. Potential diagnoses include: no abnormalities detected, myocarditis, infarction, dual pathology and pericardial inflammation. First example shows a normal 12-lead electrocardiogram (ECG) and a normal CMR study. Second example shows myocarditis, with anterolateral ST segment flattening. CMR shows normal function but patchy mid-wall enhancement in the anterior and inferior wall (red arrows) with T2 map showing co-localised oedema. The third example shows myocardial infarction with ECG anterolateral ST changes and thinning of left ventricular (LV) wall predominantly in the anterolateral segment on the short-axis image (red asterisk); corresponding LGE image shows an ischaemic pattern transmural scar (red arrow); T1 maps show elevated T1 values in the lateral wall. The fourth example shows a case of dual pathology due to myocarditis and infarction. The ECG shows hyperacute T-waves in the anterolateral leads and the CMR study shows a short-axis cine with increased signal intensity in the anterior/anteroseptal segment (red asterisk). The two LGE images reveal an ischaemic scar in this area extending from subendocardium as well as an area of sub-epicardial enhancement in the inferior wall consistent with myocarditis (red arrows). The last case presents pericardial inflammation. The ECG shows widespread concave ST-segment elevation. The 4-chamber cine is unremarkable, whereas the corresponding LGE images show pericardial enhancement (red arrows)

An elevated troponin should prompt a search for causation, and particularly imaging, as structural abnormalities are likely to be found in this cohort and further predict adverse outcomes (Fig. 1) [4, 8, 17–20, 24]. In patients with an elevated troponin and non-obstructive coronary arteries, CMR can provide the diagnosis in up to 90% of cases [25], and  is the imaging modality of choice for diagnosis of acute myocarditis [26]. The diagnostic performance of the CMR-Lake Louise imaging criteria to identify acute myocarditis has been reported to have an overall diagnostic accuracy of around 83% (sensitivity 78–80% and specificity ~ 87–88%) [27, 28]. CMR can also identify persistent inflammation and the presence of fibrosis in patients recovered from COVID-19 [9, 29], which may have long-term clinical implications. Furthermore, CMR is the reference standard method for assessing LV and RV pathology, whilst regional perfusion defects consistent with obstructive CAD can be accurately identified using CMR perfusion [30].

Based on the possibility that myocardial injury may be relatively common in COVID-19 patients, and has been shown to be a marker of poor prognosis, further studies are required to clarify the pathophysiology of SARS-CoV-2 related myocardial injury. In particular, the nature of injury may suggest the need for therapeutic intervention, which may be specific (e.g. antithrombotic, anti-inflammatory), or non-specific e.g. heart failure treatment. Importantly, the nature and extent of myocardial injury may also help risk stratify patients both acutely and chronically, and for this, multi-time-point imaging is required to separate out cause and effect. Finally, the underlying risk factors for myocardial involvement and the impact on long-term clinical outcomes need to be investigated.

## Methods

### Objectives

The primary objectives of COVID-HEART are, for a hospitalised-recovering patient population (or those recently discharged), to: (1) characterise the nature and extent of myocardial involvement in COVID-19 patients with an elevated troponin; (2) assess how cardiac involvement and clinical outcome associate with recognised risk factors for mortality in COVID-19 (age, sex, ethnicity and comorbidities) and with genetic factors; (3) evaluate if differences in myocardial recovery at 6-months are dependent on demographics, genetics and CVD comorbidities (diabetes, hypertension, heart failure and CAD/peripheral vascular disease); (4) understand the impact of recovery status at 6-months on patient-reported quality of life (QoL) and functional capacity.

The secondary objectives include: (a) to examine whether there are characteristic electrocardiogram (ECG) findings specific for SARS-CoV-2 myocarditis that are distinct from acute myocardial infarction which could minimise the requirement for invasive cardiac catheterisation and, (b) to establish the nature and prevalence of myocardial ischaemia and microvascular dysfunction (coronary vascular sub-study).

### Study design

A longitudinal, multicentre, observational cohort study of UK patients presenting with COVID-19 and elevated serum troponin, who are either recovering in hospital or were recently discharged.

### Ethics and registration

Ethical approval for the COVID-HEART study has been granted by the UK National Research Ethics Service (20/NW/0292). COVID-HEART is registered on the 'International Standard Randomised Controlled Trial’ registry (ISRCTN 5866,920) https://doi.org/10.1186/ISRCTN58667920.

### Funding, sponsorship and prioritisation

COVID-HEART is funded by the National Institute for Health Research (NIHR) and UK Research and Innovation (UKRI) COVID-19 Rapid Response Rolling Call (grant number COV0254), and sponsored by the University of Leeds, UK. The study has been endorsed by the British Society of Cardiovascular Magnetic Resonance (BSCMR) Research Group, and nationally prioritised, and received both BHF-NIHR Cardiovascular Partnership Flagship Status, and the NIHR Urgent Public Health Group identified it as an Urgent Public Health (UPH) study. Funding for the translation of the patient information leaflets into non-English languages was provided by the West Yorkshire and Humber Clinical Research Network (CV070).

### Study population

Inclusion criteria include—hospitalised-recovering patient population (age ≥ 18 years), or those recently discharged from hospital, with a diagnosis of COVID-19 based upon either a pathology or radiology diagnosis, with cardiac biomarkers (troponin I or T) increased above the sex-specific upper reference limit of the local laboratory range. Exclusion criteria include being unable or unwilling to consent, contraindication to CMR, pregnancy or breast-feeding.

Contemporary patient cohorts acting as control data will be acquired for comparative CMR analysis. These will be derived from ethically approved local research studies from the recruiting centres for COVID-HEART using the same CMR scanners (vendor and field strength). As the patients in COVID-HEART are COVID( +) and Troponin( +), control populations will include:

1. A population matched for age and CVD risk factors, who have been hospitalised and are COVID( +) and Troponin(−) [e.g. Capturing MultiORgan Effects of COVID-19 (C-MORE) cohort, Oxford, UK; NCT04510025].
2. A population matched for age and CVD risk factors, who have tested COVID(−) and Troponin(−).

### Setting

Recruitment will take place in 25 participating secondary or tertiary care hospitals across the UK, all of whom have access to well-established local CMR services.

### Recruitment process and data collection

Screening will be performed at an individual hospital level, with participating hospitals cross-referencing all admissions with a positive COVID-19 status (pathology/radiology diagnosis), with serum troponin results. This study will integrate two key aspects. Work package-1 will establish a national image repository for patients with COVID-19 who have CMR for clinical reasons. Work package-2 (objectives as outlined above) will use CMR to characterise the nature and extent of myocardial injury in a hospitalised but recovering patient population, who are serum biomarker (troponin) positive for myocardial damage.

The source data will include hospital records, National Health Service (NHS) health and social care records, clinical and office charts, laboratory and pharmacy records as well as digital images from radiology (chest X-ray/computed tomography/CMR) and cardiology (echocardiography/angiography/CMR). Patients in work package-1 who have a clinically indicated CMR and are troponin positive will be invited to join work package-2 for a follow-up CMR scan and assessment at 6 months. Clinical follow up for vital status and cardiovascular events at 12 months (and up to 5-years) will be performed by recruitment sites via local hospital electronic patient records, primary care records and by national data-linkage for other NHS databases.

Co-enrolment of patients in existing registries will be encouraged (e.g. ISARIC-4c and CAPACITY: Cardiac complicAtions in Patients with SARS Corona vIrus 2 regisTrY; NCT04325412). Also, as this is a longitudinal, observational study, co-enrollment with other UK COVID-19 studies/trials will be permitted.

### Consent procedures

All recruited patients will provide written informed consent as per the international ethical and scientific quality standard of Good Clinical Practice (GCP). Patient information sheets for COVID-HEART will be provided in English, and also in up to 10 other languages commonly spoken in the UK, including: French, Portuguese, Polish, Urdu, Bengali, Punjabi, Gujarati, Hindi, Somali and Arabic.

When eligibility criteria are confirmed, medical staff or appropriately trained support staff will seek consent from patients after allowing as much time as necessary to consider the study, or at least 24 h, whilst the patient is either an in- or out-patient. Consent for participation in the coronary vascular sub-study (ethics reference: 19/EM/0295) will be sought at the time of consent for work package-2.

All participants will have the right to withdraw from the study at any point. Moreover, the investigator may discontinue a participant from the study at any time if it is considered necessary for any reason including ineligibility, significant protocol deviation or loss to follow-up. The reason for withdrawal will be recorded. If consent is withdrawn before data are used in any research analyses, then data relating to that participant could be removed, if the participant explicitly requests that their data are not used. In general, if consent is withdrawn, or if the patient were to become incapacitated, any data collected up to that point will remain on file and will be included in the final study analysis.

### Research investigations

**Fig. 2:**
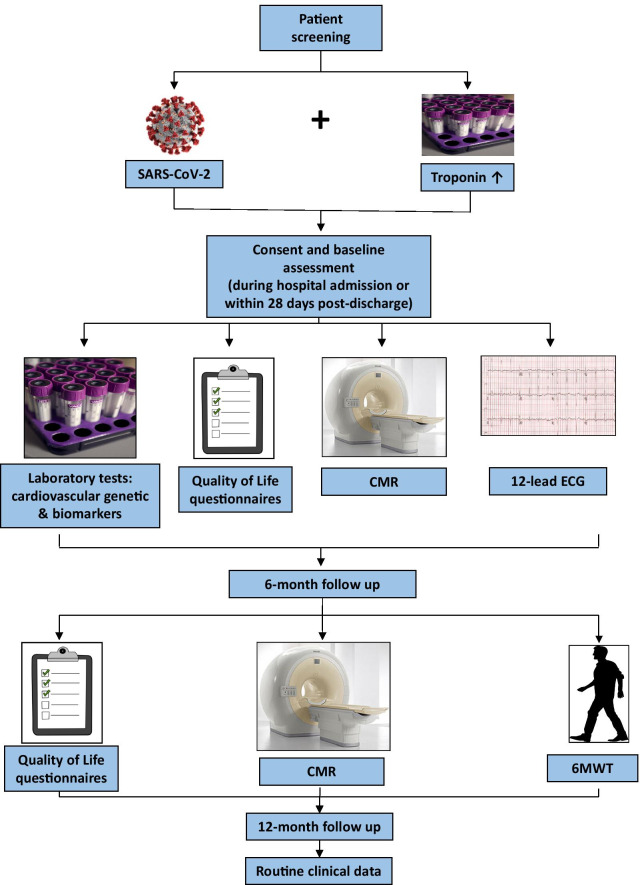
Study flow diagram. COVID-19 patients, who have an elevated troponin, will be invited to participate in the study. Patients who are recruited undergo multimodality assessment at index visit: either during their hospital admission or within 28 days of discharge. Assessment includes a 12-lead ECG, CMR study, genetic and immunological blood tests, and Quality of Life questionnaires. Patients are subsequently invited for a second visit at 6 months. At this point they undergo a repeat CMR scan, quality of life assessments and 6-min walk test. Further follow up at 12 months will be conducted via examination of routine clinical data, available through general practitioners, electronic hospital records, NICOR and NHS Digital, and in collaboration with other nationally recognised COVID-19 studies

All recruited patients will be offered CMR imaging, digital 12-lead ECG, validated quality of life questionnaires, 6 min walk test (6MWT), and additional blood sampling for genetic analysis/immunological responses/T-cell function and cardiovascular biomarkers, if not already acquired. See study flow diagram (Fig. 2) and below for detailed description of research investigations.

1)* CMR* Scans will ideally be performed pre-discharge or within 28 days of discharge, and then repeated at 6 months (± 4 weeks) from the date of admission and on the same scanner wherever possible. Scans will be performed on either a 1.5 T or a 3 T CMR system using multi-channel phased-array chest coils of any vendor, depending upon local availability. ECG gating will be employed for all cardiac gated sequences to ensure appropriate triggering. The main CMR protocol is in keeping with Society for Cardiovascular Magnetic Resonance (SCMR) recommended CMR protocols for scanning patients with active or convalescent phase COVID-19 [6], and it will take approximately 50 min to acquire, with an optional shortened and extended protocol dependent of patient preference and ability. An estimated glomerular filtration rate (eGFR) and haematocrit will be measured prior to each CMR scan. For patients with significant renal failure (eGFR < 30 ml/min/1.73 m^2^), late gadolinium enhancement (LGE) and post-contrast T1-mapping can be omitted and a contrast-free CMR scan performed.

**Fig. 3:**
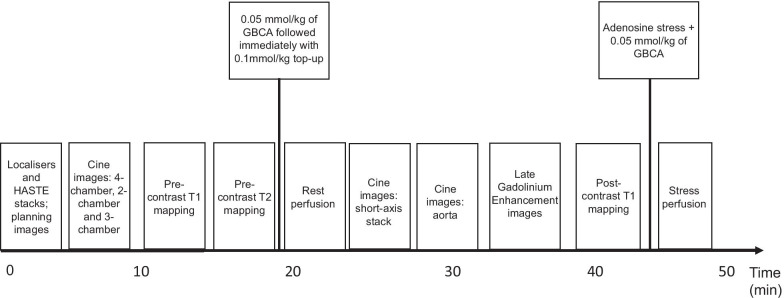
COVID-HEART study CMR protocol. The full protocol takes approximately an hour, and comprises (in order of acquisition): localisers and axial HASTE stack, cine images of the left ventricle in long-axis views, pre-contrast T1 mapping and T2 mapping, rest myocardial perfusion after injection of 0.05 mmol/kg of gadolinium based contrast agent (GBCA) followed immediately with 0.1 mmol/kg top-up (giving a total dose of 0.15 mmol/kg). Then a ventricular short-axis stack and aortic cine images (aortic distensibility, followed by late gadolinium enhancement (LGE) imaging and post-contrast T1 mapping. The last component (dependent on patient suitability) is stress perfusion imaging, performed after administration of adenosine stress at dose of 140-210mcg/kg/min, utilising a further 0.05 mmol/kg of GBCA

The main CMR study protocol includes the following components and typical parameters, which may vary by vendor and field strength, but remain comparable overall (Fig. 3):

A. Localiser sequences and breath-hold transverse Half-Fourier Acquisition Single-shot Turbo spin Echo (HASTE) imaging stacks covering lung and abdomen to 1–2 cm below the kidneys. Typical sequence parameters: TE 1.33 ms, TR 700 ms, slice thickness 8 mm, FOV = 400 mm, FOV phase 100%, flip angle 10°.
B. Cine images acquired with breath-hold balanced steady-state free precession (bSSFP) sequence. Long-axis views of the LV: 4-chamber, 2-chamber, and 3-chamber views. Sequence parameters: TE 1.05 ms, TR 40.29 ms, slice thickness 8 mm, 25% distance factor, FOV = 500 mm, FOV phase 75%, flip angle 50°.
C. Native (pre-contrast) T1-mapping: acquired using a single breath-hold shortened modified Look-Locker inversion (ShMOLLI) 5(1)1(1)1 technique [31], where available. Shimming will be performed to avoid artefacts. Native T1-mapping will be acquired in 3 short-axis cuts of the LV (basal, mid-ventricular, apical) to match the locations of segments 1–16 of the American Heart Association 17-segment model [32]. The apical segment 17 is omitted. Each acquisition will be verified against an R^2^ map (according to vendor availability). Typical pulse sequence parameters: TE 1.07 ms, TR 379 ms, slice thickness 8 mm, FOV = 360 mm (can be adjusted according to size), FOV phase 75%, flip angle 35°, distance factor 25%, generalised auto-calibrating partially parallel acquisition (GRAPPA) 2 with 24 reference lines.
D. Native (pre-contrast) T2-mapping: matching in slice location to the T1 maps, will be acquired using either a T2-prepped b-SSFP sequence with a minimum of 3 source images (e.g. MyoMaps T2-mapping for Siemens scanners), or a black-blood prepared, navigator-gated, free-breathing hybrid gradient (echo planar imaging, EPI) and spin-echo multi-echo sequence (GRASE). Typical sequence parameters: TE 1.3 ms, TR 222.43 ms, slice thickness 8 mm, FOV = 360 mm, FOV phase 80%, flip angle 20°.
E. Rest myocardial perfusion imaging: obtained using pixel-wise perfusion mapping (or locally available pulse sequence) following administration of 0.05 mmol/kg of gadolinium based contrast agent (GBCA) at 4 ml/s with 20 ml flush at 4 ml/s via a power injector. Immediately after rest perfusion image acquisition, 0.1 mmol/kg top-up (giving a total dose of 0.15 mmol/kg) of GBCA will be given. Accepted GBCA agents include: gadobutrol and gadoteric acid. Sequence parameters: TE 1.04 ms, TR 143.04 ms, slice thickness 8 mm, FOV = 380 mm, FOV 75%, flip angle 50°.
F. Ventricular short-axis stack. Sequence parameters will match the cine image acquisition in long-axis.
G. Aortic distensibility imaging: planned using bSSFP cine sequence of the thoracic aorta in sagittal oblique view and then acquired transverse to the ascending/descending thoracic aorta (at pulmonary artery bifurcation level). Blood pressure will be recorded whilst in the scanner to allow calculation of aortic distensibility. Sequence parameters: TE 1.25 ms, TR 43.35 ms, slice thickness 6 mm, FOV = 380 mm, FOV phase 100%, flip angle 55°, 50 phases.
H. LGE images will be acquired ~ 5–15 min after intravenous injection of 0.1–0.15 mmol/kg of GBCA with a free-breathing phase-sensitive motion correction bSSFP or breath-hold, segmented inversion-recovery sequence. Contiguous stack of LV short-axis images and single long-axis slices in 2-chamber, 4-chamber and 3-chamber at the same slice locations as obtained for cine imaging will be acquired. A Look-Locker sequence will be used to determine the appropriate inversion time (TI) [33]. Example parameters are: TI will be adjusted as per Look-Locker sequence, TE 1.14 ms, TR 411.48 ms, matrix 128 × 256, 8 mm slice thickness with 2 mm inter-slice gap, FOV = 400 mm, FOV phase 75%, flip angle 47°.
I. Post-contrast T1 measurements will be acquired at the exact same locations as the native T1-maps and performed at least 10 min after injection of GBCA, using the same pulse sequence and parameters as the native T1-maps.
J. A subset of patients eligible for coronary vascular assessment will undergo an extended protocol at baseline to include stress-perfusion imaging. Adenosine stress perfusion imaging will be acquired after post-contrast T1 mapping using an adenosine dose of 140–210 mcg/kg/min for 3–5 min, whilst monitoring symptoms, heart rate and blood pressure to assess an adequate response. Subsequently, GBCA will be given at a dose of 0.05 mmol/kg at 4 ml/s followed by a 20 ml flush at 4 ml/s, giving a total dose of 0.2 mmol/kg. Stress perfusion imaging will be acquired in the same slice locations as the rest perfusion images. Sequence parameters will match the resting scan.

2)* Digital 12-lead ECG.* A 12-lead ECG will be performed either as part of routine clinical assessment or as an additional research test during the index admission and again at 6 months follow up. In selected centres (which will be provided with an identical study-specific digital ECG machine), a full 12-lead digital ECG from COVID-19 patients will be recorded daily, if possible dependent on clinical status, for up to 7 days, whilst an in-patient. Digital ECGs in patients with CMR-proven myocarditis will be compared to a reference dataset of acute ST-elevation myocardial infarction patients already available from the NIHR-Efficacy and Mechanism Evaluation Programme funded T-TIME randomised controlled trial [34].

3)* Functional assessment by 6-min walk test.* Participants will asked to perform a standard 6MWT at the 6-month visit following the American Thoracic Society guidelines [35]. Patients will be instructed to walk along a corridor and turn at 15 m in order to cover the maximum distance in 6 min under the supervision of study investigators. The investigators will tell the participant how much time had elapsed every 2 min and encourage participants to continue at intervals of between 30 s and 1 min. At the end of 6 min, participants will be asked to stop, and the distance walked will be measured in metres.

4)* Quality of life questionnaires.* All participants will be asked to complete patient-reported health status questionnaires (36-Item Short Form Health Survey; SF-36v2) and a health-related QoL questionnaire (EQ-5D-5L) at baseline and 6 months. These will be performed either face to face, by telephone or by post, depending on local site and individual patient circumstances and preferences. These questionnaires were chosen to align with other UK national COVID-19 research studies (e.g. C-MORE/P-HOSP-COVID).

5)* Laboratory investigations*. As part of clinical routine, patients will undergo routine laboratory testing, including full blood count, renal and liver profile, inflammatory markers, and cardiovascular biomarkers (e.g. troponin and brain natriuretic peptide (BNP)). Additional research blood sampling for genetic analysis/inflammatory markers will be performed, if not already acquired from participation in other UK COVID-19 research studies. 10 ml of blood will be drawn into an ethylenediaminetetraacetic acid (EDTA) bottle. The tube will be inverted 5 times and transferred on ice. The sample will be centrifuged for 20 min at 2000G at 4 °C. The sample will be divided into 6 × 1 ml Thermo Matrix tubes. Subsequently a pipette will be used to provide 5 aliquots. Plasma will be taken from the centre of each sample avoiding the buffy coat layer, which will be retained separately. Samples will be labelled with a unique study ID and stored locally on-site, pending batch transfer for analysis at Leicester University and the University of Glasgow.

### Follow-up

Patients will be followed up at 6 months (± 4 weeks) from date of the diagnosis of COVID-19, and invited to have a second CMR scan, ECG, QoL assessment (SF-36v2 and EQ-5D-5L) and functional assessment (6MWT). In addition, routine clinical outcome data will be collated up to 5 years post-infection from: electronic patient records, general practice information systems, National Institute for Cardiovascular Outcomes Research (NICOR) and NHS Digital (eDRIS in Scotland).

### Study outcomes

#### Main outcomes

1. Presence, extent, distribution of LGE
2. CMR biventricular volumes and function
3. Functional capacity (6MWT) and QoL measures
4. Long-term clinical outcomes

#### Exploratory outcomes

1. CMR: T1, T2 mapping indices, myocardial strain, myocardial perfusion, aortic distensibility measures.
2. ECG indices
3. Biomarker and genetic analyses
4. Coronary atheroma extent (coronary vascular sub-study)

### Analysis plan

The prevalence and extent of cardiac involvement in patients with COVID-19 and raised troponin will be described by CMR. Predictors of cardiac dysfunction and recovery such as: blood biomarkers, the severity of the acute infection, genetics, and comorbidities will be identified. The determinants of adverse clinical outcome in this population will also be examined. Outcome assessments (clinical event adjudication) will be undertaken at individual hospital sites according a pre-specified standard definition set (Appendix 1) contained with a signed clinical outcome charter.

1)* CMR data:* All quantitative analyses will be performed blinded to participant status. CMR data sets will be analysed by a disseminated core-lab technique as previously used in the BSCMR UK valve consortium [36]. LV structure and function including mass and wall thickness will be analyzed using a clinically validated artificial intelligence (AI) analysis platform [37]. Left atrial area and global longitudinal shortening will be similarly analyzed by further validated artificial intelligence approaches [38]. LGE patterns will also be classified into patterns reflecting likely aetiology and quantitative assessment will be performed by semi-automated signal intensity analysis according to the full width at half maximum technique and other thresholds where appropriate [36]. Evidence of pericarditis will be assessed on cine, LGE and tissue characterisation images. T1 and T2 mapping will be quality-assured and analysed to establish the global and segmental T1 and T2 values, using standardised approaches developed by the Oxford group [39, 40]. Myocardial perfusion assessment for the coronary vascular sub-study will be carried out by segmental and global quantitative myocardial blood flow calculation during rest and adenosine stress; it will be calculated from fully automated in-line quantification [41, 42]. In addition, visual perfusion defects will be scored semi-quantitatively by 2 observers as previously described [43] with disagreement resolved by a third reader. The likelihood of microvascular disease (patchy perfusion defects not following the distribution of a coronary distribution or circumferential defects not affecting apical segments) will be recorded. Circumferential strain and strain rates will be generated by automated tissue tracking software (cvi42, Circle Cardiovascular Imaging, Alberta, Calgary, Canada) as previously described [44]. Aortic distensibility will be assessed using blood pressure assessments and measurements of the cross-sectional area of the ascending aorta in systole and diastole on axial cine slices at the level of the right pulmonary artery. Distensibility = (aortic max lumen area − aortic min lumen area)/(aortic min lumen area * [systolic blood pressure − diastolic blood pressure]).

2)* ECG data:* The standard and digital 12-lead ECGs will be electronically transferred via the study portal into the image repository at the coordinating centre in Glasgow, Scotland. Digital ECGs will be analysed in the University of Glasgow ECG core lab utilising the previously validated Glasgow ECG analysis software [45]. A full set of measurements plus interpretation will be extracted for analysis of ECG changes and statistical processing, with the aim of determining sensitive and specific criteria for diagnosing myocarditis from the ECG, and prognostic ECG findings in this population [46]. ECGs available in printed form will also be electronically transmitted in pdf format to the ECG Core Lab for standardised review based on the same criteria as used in the Glasgow program. Manually classified changes will also be subject to statistical analysis.

3)* Laboratory data:* All blood samples will be transferred to the central coordinating centre in Leicester, England and analysed in the University of Leicester Cardiovascular Research Centre and the BHF Glasgow Cardiovascular Research Centre, University of Glasgow. Analysis will entail novel cardiovascular biomarkers (e.g. vascular cell adhesion molecule, interleukin-6) and markers of genetic susceptibility to COVID-19. The genetic analysis will be used to explore links specifically in the black and minority ethnic populations and linked in with existing cohorts for genotyping (The Genetics and Vascular Health Check study, The Biomedical Research Informatics Centre for Cardiovascular Science and UK Biobank).

4)* 6MWT:* As per the American Thoracic Society guidelines, the distance walked (measured in meters) in 6 min will be recorded by the investigator supervising the test. Symptoms of perceived breathlessness will be recorded on the Borg scale and if the test was stopped early, the reason will be recorded.

5)* Quality of life questionnaires:* Patients’ responses to the 36-Item Short Form Health Survey and the health-related QoL questionnaire (EQ-5D-5L) will be recorded in the eCRF by the investigators. Each of the 8 health domains measured by the SF-36v2 will be given a combined percentage score, with higher percentage scores representing a better level of functioning. Each of the 5 domains of the EQ-5D-5L will be scored on a 1–5 scale, with level 1 indicating no problems with a particular domain and level 5 indicating extreme problems. Health state of an individual patient will be determined by combining the levels from all the domains.

### Statistical considerations

#### Sample size considerations

Patients in work package-1 will originate from the open national image repository for all UK CMR centres, and therefore no sample size calculation is required. For work package-2, based on the assumption that the mean prevalence of myocardial involvement was 12% (mid-range) from previous studies, a precision of 3.5% and 95% confidence level, a sample size of 333 ± 3.5% would be required. To account for a 10% drop out rate, it was calculated that 370 patients are needed.

### Statistical analysis

Statistical analyses will be conducted after production of a signed statistical analysis plan and once baseline and follow-up data collection has been completed. Broadly, the statistical methods for each study objective will be as follows:

1. to characterise the nature and extent of myocardial involvement in COVID-19 patients with an elevated troponin, analyses will be descriptive, and will involve estimation of prevalence with 95% confidence intervals (CIs);
2. to assess how cardiac involvement and clinical outcomes associate with recognised risk factors for mortality in COVID-19 (age, sex, ethnicity and comorbidities) and with genetic factors, we shall present tabular and graphical descriptive summaries within subgroups, and use logistic regression to test for associations and provide estimates with 95% CIs;
3. to evaluate if differences in myocardial recovery at 6 months are associated with demographics, genetics and CV comorbidities (diabetes, hypertension, heart failure and CAD/peripheral vascular disease), descriptive summaries, and linear and logistic regression methods will be applied;
4. to understand the association between recovery status at 6 months and patient-reported QoL and functional capacity, descriptive methods, and linear regression will be used.

To assess whether there are characteristic ECG findings specific for SARS-CoV-2 myocarditis, measures of diagnostic performance will be calculated, and ROC analyses will be used.

In the coronary vascular sub-study, descriptive methods will be used to establish the nature and prevalence of myocardial ischaemia and microvascular dysfunction, and linear regression methods, including mixed effects methods for modelling myocardial blood flow within segments 1–16 of the American Heart Association 17-segment model, will be used to assess risk factors.

Throughout these analyses, levels of missing data will be reported, and regression models will be fitted using multiple imputation of missing predictor variables. Where appropriate, data from other cohorts will be used as control populations.

### Data monitoring and linkage

The University of Glasgow will be coordinating centre for data and analysis and will hold identifiable data in secured databases to permit record linkage. The Universities of Leicester and Glasgow and NHS Laboratory Medicine (including the Biorepository) will undertake storage and/or analysis of blood samples (DNA, RNA, and small molecules).

As part of the patient consent process, it will be explicitly requested that anonymised data and images can be shared with other national and global research initiatives, for efficiency in data collection and sharing across other nationally prioritised UK COVID studies (e.g. C-MORE, P-HOSP-COVID and CISCO-19 [Cardiovascular and Pulmonary Imaging in SARS Coronavirus disease-19]) [47], as well as with other international CMR studies and registries e.g. the international SCMR COVID-19 registry. The principles set out in the 2016 Statement on data sharing in public health emergencies will be followed and it will be ensured that the World Health Organization (WHO) has rapid access to the emerging findings that could aid the global response.

As this is an observational study only serious adverse events (SAEs) that relate directly to the participation in the study procedures and sample collection will be reported to the sponsor and Ethics committee. All hospitalisations and deaths will be recorded as study outcomes.

The management of incidental findings of the research CMR scans will be performed at the individual site level according to the normal local practice and procedures.

## Discussion

The COVID-HEART study is a longitudinal, multicentre, observational cohort study of UK patients presenting with COVID-19 infection and positive troponin, who are either recovering in hospital or were recently discharged. It aims to utilise CMR in evaluation of the nature and extent of myocardial injury and subsequently, myocardial recovery in these patients, and to examine the impact of myocardial recovery status on quality of life and functional capacity. Additional investigations include laboratory testing, which will encompass routine tests, novel cardiac biomarkers, and genetic analysis. These results will be shared with other global initiatives to address genetic susceptibility to COVID-19. Furthermore, ECGs will be investigated with the aim of identifying sensitive and specific changes that would allow diagnosis of myocarditis without the need for invasive testing.

As COVID-19 is an unprecedented global health emergency and previous studies showed that a significant proportion of patients had myocardial injury, it is important to further understand the underlying pathophysiology, risk factors and long-term outcomes. Therefore, the results of the COVID-HEART study, and the results of other registries examining myocardial injury (e.g. PHOSP-COVID, ISRCTN10980107; MOIST, NCT04525404; MYOCOVID, NCT04375748; MIIC-MI, NCT04412369; CARDOVID, NCT04455347; CISCO-19, NCT04403607), may have significant clinical implications on the assessment and management of these patients. Furthermore, the new knowledge from these studies will help inform disease-targeted therapy development, for the prevention and treatment of cardiovascular complications in COVID-19.

## Data Availability

The datasets used and/or analysed during the current study will be available from the corresponding author on reasonable request. Anonymised data may be shared with other COVID-19 global initiatives.

## Appendix 1: Summary definitions of clinical outcomes

A pre-specified, signed, *Clinical Outcomes Charter* will be provided to each recruitment centre. This will contain detailed endpoint definitions to facilitate consistent local adjudication and unbiased reporting. Endpoint definitions will align with the 2014 American College of Cardiology/American Heart Association Key Data Elements and Definitions for Cardiovascular Endpoint Events in Clinical Trials [48]. In brief, these endpoints will include:

1. *Cardiovascular death* is a defined as death occurring secondary to an acute myocardial infarction, sudden cardiac death, heart failure, stroke, death resulting from cardiovascular procedures or death due to cardiovascular haemorrhage.
2. *Non-cardiovascular death* is defined as any death with a specific cause that is not thought to be due to a cardiovascular cause. There should be unequivocal and documented evidence of a non-cardiovascular cause of death.
3. *Myocardial infarction* is defined as elevation of cardiac biomarkers above the 99th percentile of the upper reference limit in the setting of an appropriate clinical presentation in combination with diagnostic ischaemic electrocardiographic changes. Specific clinical classification of different types of myocardial infarction will be assigned from the 4th Universal Definition of Myocardial Infarction [12].
4. *Coronary artery revascularisation*, either by percutaneous coronary intervention (PCI) or coronary artery bypass graft surgery (CABG).
5. *Transient ischaemic attack* is defined as an episode of focal neurological dysfunction resulting from brain, retinal and spinal cord ischaemia, that is transient in nature and resolves within 24 h.
6. *Stroke* is defined as a focal or global neurological dysfunction secondary to brain, retinal or spinal cord injury resulting from haemorrhage or infarction.
7. *Pulmonary embolism* is defined based upon clinical evaluation and a diagnostic imaging test, ideally, computed tomography (CT) pulmonary angiography. If ventilation/perfusion scintigraphy is used, then the diagnosis may be accepted based on clinical evaluation and a high probability scan. The clinical evaluation and diagnosis of pulmonary embolism should align with the 2019 ESC Guidelines for the diagnosis and management of acute pulmonary embolism [49].
8. *Deep venous thrombosis (DVT)* diagnosis should be established based on a clinical evaluation, D-dimer elevation and imaging findings (duplex ultrasound or CT angiography).
9. *Myopericarditis* diagnosis will be adjudicated based upon the 2013 Position Statement of the European Society of Cardiology Working Group on Myocardial and Pericardial Diseases.(50)
10. *Hospitalisation for other cardiovascular cause*:
  - *Heart failure* events are defined as hospitalisation for heart failure or an urgent outpatient visit. A heart failure hospitalisation event must fulfil the following criteria: primary admission diagnosis is heart failure, the patient experiences either worsening of heart failure symptoms or a new symptom, and requires an increase in therapy.
  - *Arrhythmia* defined as hospitalisation with an arrhythmia (atrial, supraventricular and/or ventricular or brady-arrhythmia) documented by electrocardiography (single- or multi-lead) with a change in treatment (withhold, change in dose of existing medication, prescription of new medication for arrhythmia) and/or pacemaker therapy.
  - *Chest pain/unstable angina* defined as an emergency/unplanned admission to a hospital setting that results in at least one overnight stay (i.e. a date change) with cardiac ischaemic-type symptoms at rest or minimal exertion.

## Abbreviations

6MWT: Six minute walk test
BNP: Brain natriuretic peptide
BSCMR: British Society of Cardiovascular Magnetic Resonance
bSSFP: Balanced steady state free precession
C-MORE: Capturing Multi-ORgan Effects of COVID-19
CAD: Coronary artery disease
CMR: Cardiovascular magnetic resonance
COVID-19: Coronavirus disease 2019
CVD: Cardiovascular disease
ECG: Electrocardiogram
eCRF: Electronic case report form
ECV: Extracellular volume fraction
EDTA: Ethylenediaminetetraacetic acid
eGFR: Estimated glomerular filtration rate
EPI: Echo planar imaging
FBC: Full blood count
FOV: Field-of-view
GBCA: Gadolinium-based contrast agent
GCP: Good clinical practice
GRAPPA: Generalised auto-calibrating partially parallel acquisition
GRE: Gradient recalled echo
HASTE: Half-Fourier acquisition single-shot turbo spin echo
LGE: Late gadolinium enhancement
LV: Left ventricle/left ventricular
LVOT: Left ventricular outflow tract
NHS: National Health Service
NICOR: The National Institute for Cardiovascular Outcomes Research
P-HOSP-COVID: Post-hospitalisation COVID-19 study
QoL: Quality of life
RNA: Ribonucleic acid
RV: Right ventricle/right ventricular
SAEs: Serious adverse events
SARS-CoV-2: Severe acute respiratory syndrome coronavirus 2
SCMR: Society for Cardiovascular Magnetic Resonance
SF-36: 36-Item Short Form Health Survey
ShMOLLI: Shortened Modified Look-Locker Inversion recovery
TE: Echo time
TI: Inversion time
TR: Repetition time
NIHR-UKRI: National Institute for Health Research—UK Research Institute
VLA: Vertical long axis
WHO: World Health Organization

## References

[1] Bandyopadhyay D, Akhtar T, Hajra A, Gupta M, Das A, Chakraborty S (2020). COVID-19 pandemic: cardiovascular complications and future implications. Am J Cardiovasc Drugs.

[2] Gavriatopoulou M, Korompoki E, Fotiou D, Ntanasis-Stathopoulos I, Psaltopoulou T, Kastritis E (2020). Organ-specific manifestations of COVID-19 infection. Clin Exp Med..

[3] Raman B, Cassar MP, Tunnicliffe EM, Filippini N, Griffanti L, Alfaro-Almagro F (2020). Medium-term effects of SARS-CoV-2 infection on multiple vital organs, exercise capacity, cognition, quality of life and mental health, post-hospital discharge. EClinicalMedicine.

[4] Bansal M (2020). Cardiovascular disease and COVID-19. Diabetes Metab Syndr.

[5] Aghagoli G, Gallo Marin B, Soliman LB, Sellke FW (2020). Cardiac involvement in COVID-19 patients: risk factors, predictors, and complications: a review. J Cardiac Surg..

[6] Kelle S, Bucciarelli-Ducci C, Judd RM, Kwong RY, Simonetti O, Plein S (2020). Society for Cardiovascular Magnetic Resonance (SCMR) recommended CMR protocols for scanning patients with active or convalescent phase COVID-19 infection. J Cardiovasc Magn Reson.

[7] Clerkin Kevin J, Fried Justin A, Raikhelkar J, Sayer G, Griffin Jan M, Masoumi A (2020). COVID-19 and cardiovascular disease. Circulation.

[8] Giustino G, Croft Lori B, Stefanini Giulio G, Bragato R, Silbiger Jeffrey J, Vicenzi M (2020). Characterization of myocardial injury in patients with COVID-19. J Am Coll Cardiol.

[9] Puntmann VO, Carerj ML, Wieters I, Fahim M, Arendt C, Hoffmann J (2020). Outcomes of cardiovascular magnetic resonance imaging in patients recently recovered from coronavirus disease 2019 COVID-19. JAMA Cardiol..

[10] Libby P (2020). The heart in COVID-19. JACC Basic Transl Sci..

[11] Song Y, Gao P, Ran T, Qian H, Guo F, Chang L (2020). High Inflammatory burden: a potential cause of myocardial injury in critically ill patients with COVID-19. Front Cardiovasc Med.

[12] Thygesen K, Alpert JS, Jaffe AS, Chaitman BR, Bax JJ, Morrow DA (2019). Fourth universal definition of myocardial infarction (2018). Eur Heart J.

[13] Klok FA, Kruip M, Van Der Meer N, Arbous M, Gommers D, Kant K (2020). Confirmation of the high cumulative incidence of thrombotic complications in critically ill ICU patients with COVID-19: an updated analysis. Thromb Res..

[14] Pellegrini D, Kawakami R, Guagliumi G, Sakamoto A, Kawai K, Gianatti A (2021). Microthrombi as a major cause of cardiac injury in COVID-19: a pathologic study. Circulation.

[15] Stefanini GG, Montorfano M, Trabattoni D, Andreini D, Ferrante G, Ancona M (2020). ST-elevation myocardial infarction in patients with COVID-19: clinical and angiographic outcomes. Circulation.

[16] Cosyns B, Lochy S, Luchian ML, Gimelli A, Pontone G, Allard SD (2020). The role of cardiovascular imaging for myocardial injury in hospitalized COVID-19 patients. Eur Heart J Cardiovasc Imaging.

[17] Karbalai Saleh S, Oraii A, Soleimani A, Hadadi A, Shajari Z, Montazeri M (2020). The association between cardiac injury and outcomes in hospitalized patients with COVID-19. Intern Emerg Med.

[18] Nie S-F, Yu M, Xie T, Yang F, Wang H-B, Wang Z-H (2020). Cardiac Troponin I is an independent predictor for mortality in hospitalized patients with COVID-19. Circulation.

[19] Mitrani RD, Dabas N, Goldberger JJ (2020). COVID-19 cardiac injury: Implications for long-term surveillance and outcomes in survivors. Heart Rhythm.

[20] Lala A, Johnson Kipp W, Januzzi James L, Russak Adam J, Paranjpe I, Richter F (2020). Prevalence and impact of myocardial injury in patients hospitalized with COVID-19 infection. J Am Coll Cardiol.

[21] Zhou F, Yu T, Du R, Fan G, Liu Y, Liu Z (2020). Clinical course and risk factors for mortality of adult inpatients with COVID-19 in Wuhan, China: a retrospective cohort study. Lancet.

[22] Li B, Yang J, Zhao F, Zhi L, Wang X, Liu L (2020). Prevalence and impact of cardiovascular metabolic diseases on COVID-19 in China. Clin Res Cardiol.

[23] Novel CPERE (2020). The epidemiological characteristics of an outbreak of 2019 novel coronavirus diseases (COVID-19) in China. Zhonghua Liu Xing Bing Xue Za Zhi.

[24] Sandoval Y, Januzzi JL, Jaffe AS (2020). Cardiac troponin for assessment of myocardial injury in COVID-19: JACC review topic of the week. J Am Coll Cardiol.

[25] Emrich T, Emrich K, Abegunewardene N, Oberholzer K, Dueber C, Muenzel T (2015). Cardiac MR enables diagnosis in 90% of patients with acute chest pain, elevated biomarkers and unobstructed coronary arteries. Br J Radiol.

[26] Ponikowski P, Voors AA, Anker SD, Bueno H, Cleland JG, Coats AJ (2016). 2016 ESC Guidelines for the diagnosis and treatment of acute and chronic heart failure: The Task Force for the diagnosis and treatment of acute and chronic heart failure of the European Society of Cardiology (ESC) developed with the special contribution of the Heart Failure Association (HFA) of the ESC. Eur Heart J.

[27] Friedrich MG, Sechtem U, Schulz-Menger J, Holmvang G, Alakija P, Cooper LT (2009). Cardiovascular magnetic resonance in myocarditis: a JACC White Paper. J Am Coll Cardiol.

[28] Ferreira VM, Schulz-Menger J, Holmvang G, Kramer CM, Carbone I, Sechtem U (2018). Cardiovascular magnetic resonance in nonischemic myocardial inflammation: expert recommendations. J Am Coll Cardiol.

[29] Huang L, Zhao P, Tang D, Zhu T, Han R, Zhan C (2020). Cardiac involvement in patients recovered from COVID-2019 identified using magnetic resonance imaging. JACC Cardiovasc Imaging..

[30] Plein S, Kozerke S, Suerder D, Luescher TF, Greenwood JP, Boesiger P (2008). High spatial resolution myocardial perfusion cardiac magnetic resonance for the detection of coronary artery disease. Eur Heart J.

[31] Piechnik S, Ferreira VM, Dall’Armellina E, Cochlin LE, Greiser A, Neubauer S, Robson MD (2010). Shortened Modified Look-Locker Inversion recovery (ShMOLLI) for clinical myocardial T1-mapping at 1.5 and 3 T within a 9 heartbeat breathhold. J Cardiovasc Magn Reson..

[32] Cerqueira MD (2002). American Heart Association Writing Group on Myocardial Segmentation and Registration for Cardiac Imaging: standardized myocardial segmentation and nomenclature for tomographic imaging of the heart: a statement for healthcare professionals from the Cardiac Imaging Committee of the Council on Clinical Cardiology of the American Heart Association. Circulation.

[33] Look DC, Locker DR (1970). Time saving in measurement of NMR and EPR relaxation times. Rev Sci Instrum.

[34] Maznyczka AM, McCartney PJ, Eteiba H, Greenwood JP, Muir DF, Chowdhary S (2020). One-year outcomes after low-dose intracoronary alteplase during primary percutaneous coronary intervention: the T-TIME randomized trial. Circ Cardiovasc Interv..

[35] American TS (2002). ATS statement: guidelines for the six-minute walk test. Am J Respir Crit Care Med.

[36] Musa TA, Treibel TA, Vassiliou VS, Captur G, Singh A, Chin C (2018). Myocardial scar and mortality in severe aortic stenosis: data from the BSCMR valve consortium. Circulation.

[37] Augusto JB, Davies RH, Bhuva AN, Knott KD, Seraphim A, Alfarih M (2020). Diagnosis and risk stratification in hypertrophic cardiomyopathy using machine learning wall thickness measurement: a comparison with human test-retest performance. Lancet Digit Health.

[38] Maceira AM, Cosín-Sales J, Roughton M, Prasad SK, Pennell DJ (2010). Reference left atrial dimensions and volumes by steady state free precession cardiovascular magnetic resonance. J Cardiovasc Magn Reson.

[39] Carapella V, Puchta H, Lukaschuk E, Marini C, Werys K, Neubauer S (2020). Standardized image post-processing of cardiovascular magnetic resonance T1-mapping reduces variability and improves accuracy and consistency in myocardial tissue characterization. Int J Cardiol.

[40] Zhang Q, Werys K, Popescu IA, Biasiolli L, Ntusi NAB, Desai M (2021). Quality assurance of quantitative cardiac T1-mapping in multicenter clinical trials—a T1 phantom program from the hypertrophic cardiomyopathy registry (HCMR) study. Int J Cardiol..

[41] Kellman P, Hansen MS, Nielles-Vallespin S, Nickander J, Themudo R, Ugander M (2017). Myocardial perfusion cardiovascular magnetic resonance: optimized dual sequence and reconstruction for quantification. J Cardiovasc Magn Reson.

[42] Knott KD, Seraphim A, Augusto JB, Xue H, Chacko L, Aung N (2020). The prognostic significance of quantitative myocardial perfusion: an artificial intelligence–based approach using perfusion mapping. Circulation.

[43] Hussain ST, Paul M, Plein S, McCann GP, Shah AM, Marber MS (2012). Design and rationale of the MR-INFORM study: stress perfusion cardiovascular magnetic resonance imaging to guide the management of patients with stable coronary artery disease. J Cardiovasc Magn Reson.

[44] Graham-Brown M, Gulsin G, Parke K, Wormleighton J, Lai F, Athithan L (2019). A comparison of the reproducibility of two cine-derived strain software programmes in disease states. Eur J Radiol.

[45] Macfarlane PW, Devine B, Clark E (2005). The University of Glasgow (Uni-G) ECG analysis program. Comput Cardiol..

[46] Fischer K, Marggraf M, Stark AW, Kaneko K, Aghayev A, Guensch DP (2020). Association of ECG parameters with late gadolinium enhancement and outcome in patients with clinical suspicion of acute or subacute myocarditis referred for CMR imaging. PLoS ONE.

[47] Mangion K, Morrow A, Bagot C, Bayes H, Blyth KG, Church C (2020). The chief scientist office cardiovascular and pulmonary imaging in SARS Coronavirus disease-19 (CISCO-19) study. Cardiovasc Res.

[48] Hicks KA, Mahaffey KW, Mehran R, Nissen SE, Wiviott SD, Dunn B (2018). 2017 Cardiovascular and stroke endpoint definitions for clinical trials. Circulation.

[49] Konstantinides SV, Meyer G, Becattini C, Bueno H, Geersing G-J, Harjola V-P (2020). 2019 ESC Guidelines for the diagnosis and management of acute pulmonary embolism developed in collaboration with the European Respiratory Society (ERS) The Task Force for the diagnosis and management of acute pulmonary embolism of the European Society of Cardiology (ESC). Eur Heart J.

[50] Caforio AL, Pankuweit S, Arbustini E, Basso C, Gimeno-Blanes J, Felix SB (2013). Current state of knowledge on aetiology, diagnosis, management, and therapy of myocarditis: a position statement of the European Society of Cardiology Working Group on Myocardial and Pericardial Diseases. Eur Heart J.

